# Human Fecal Metabolome Reflects Differences in Body Mass Index, Physical Fitness, and Blood Lipoproteins in Healthy Older Adults

**DOI:** 10.3390/metabo11110717

**Published:** 2021-10-21

**Authors:** Mengni Cui, Alessia Trimigno, Josue L. Castro-Mejía, Søren Reitelseder, Jacob Bülow, Rasmus Leidesdorff Bechshøft, Dennis Sandris Nielsen, Lars Holm, Søren Balling Engelsen, Bekzod Khakimov

**Affiliations:** 1 Chemometrics and Analytical Technology Section, Department of Food Science University of Copenhagen Rolighedsvej 26, 1958 Frederiksberg C, Denmark; mengni@food.ku.dk (M.C.); alessia@food.ku.dk (A.T.); 2 Food Microbiology & Fermentation Section, Department of Food Science University of Copenhagen Rolighedsvej 26, 1958 Frederiksberg C, Denmark; jcame@food.ku.dk (J.L.C.-M.); dn@food.ku.dk (D.S.N.); 3 Institute of Sports Medicine, Department of Orthopedic Surgery, Bispebjerg and Frederiksberg Hospital, Nielsine Nielsens Vej 11, 2400 Copenhagen, Denmark; s.reitelseder@gmail.com (S.R.); jacob.buelow.02@regionh.dk (J.B.); r.bechshoeft@gmail.com (R.L.B.); L.Holm@bham.ac.uk (L.H.); 4 School of Sport, Exercise and Rehabilitation Sciences, University of Birmingham, Edgbaston, Birmingham B15 2TT, UK

**Keywords:** human fecal metabolome, BMI, fitness, lipoproteins, ^1^H NMR, GC-MS, SCFA, Signature Mapping

## Abstract

This study investigated how body mass index (BMI), physical fitness, and blood plasma lipoprotein levels are related to the fecal metabolome in older adults. The fecal metabolome data were acquired using proton nuclear magnetic resonance spectroscopy and gas chromatography–mass spectrometry on 163 healthy older adults (65–80 years old, 80 females and 83 males). Overweight and obese subjects (BMI ≥ 27) showed higher levels of fecal amino acids (AAs) (valine, alanine, and phenylalanine) compared to normal-weight subjects (BMI ≤ 23.5). Adults classified in the high-fitness group displayed slightly lower concentrations of fecal short-chain fatty acids, propionic acid, and AAs (methionine, leucine, glutamic acid, and threonine) compared to the low-fitness group. Subjects with lower levels of cholesterol in low-density lipoprotein particles (LDL*chol,* ≤2.6 mmol/L) displayed higher fecal levels of valine, glutamic acid, phenylalanine, and lactic acid, while subjects with a higher level of cholesterol in high-density lipoprotein particles (HDL*chol,* ≥2.1 mmol/L) showed lower fecal concentration of isovaleric acid. The results from this study suggest that the human fecal metabolome, which primarily represents undigested food waste and metabolites produced by the gut microbiome, carries important information about human health and should be closely integrated to other *omics* data for a better understanding of the role of the gut microbiome and diet on human health and metabolism.

## 1. Introduction

Human fecal matter is a waste product of food digestion and mainly contains water, undigested food remnants, including nitrogen and protein matter, carbohydrates, lipids, and hundreds of small molecular metabolites, as well as bacteria [1]. Fecal matter is also a rich source of metabolic information and the noninvasive sampling has increased interest toward exploring the human fecal metabolome as a function of diet, diseases, and other lifestyle conditions [1,2]. Recent studies have shown close relationships between the dynamics of the fecal metabolome and gut microbiome functions [3,4]. Numerous studies have related changes in the fecal metabolome with chronic and genetic diseases and linked them with the gut microbiome functions [5]. The impacts of various chronic diseases on the human fecal metabolome, including bowel disease [6], obesity [7], type-2 diabetes (T2D) [8], liver diseases [9,10], and cardiovascular diseases (CVDs) [11], have shown the potentiality of fecal metabolomics for better understanding of the pathologies of these diseases. There also evidence suggesting that the majority of the above-mentioned chronic diseases are largely influenced by BMI [12], lifestyles (e.g., diet, exercise) [13], physical fitness [14], and clinical blood parameters such as plasma concentrations of lipoproteins (LPs) [15].

**Figure 1:**
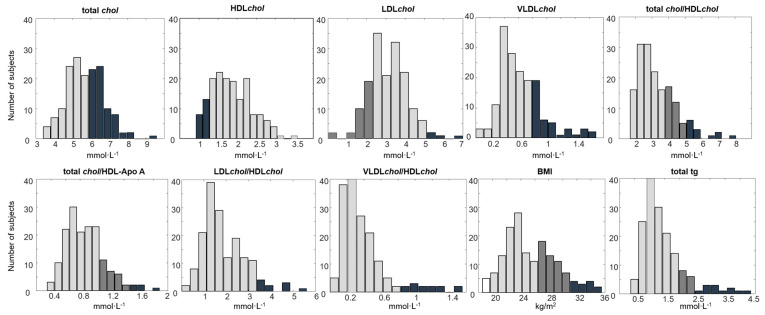
Histograms showing the distributions of human blood lipoproteins (LPs), their ratios, and BMI across the 163 subjects included in this study. The four colors in the figure represent four levels of risk thresholds set in the Danish healthcare system [21], including below the normal range (white), normal range (light grey), borderline range (dark grey), and under risk (black). The unit for LP levels and total tg is mmol/L, and that for BMI is kg/m^2^. BMI = body mass index, HDL*chol* = high-density lipoprotein cholesterol, VLDL*chol* = very-low-density lipoprotein cholesterol, LDL*chol* = low-density lipoprotein cholesterol, total tg= total triglycerides, total *chol* = total cholesterol and total *chol*/HDL*chol*, HDL-Apo A = high-density lipoprotein-Apolipoprotein A.

This paper reports a fecal metabolomics study performed on healthy older adults using proton (^1^H) nuclear magnetic resonance (NMR) spectroscopy [16] and untargeted gas chromatography-mass spectrometry (GC-MS) [17]. In addition, lipoprotein profiling of plasma samples was performed using ^1^H NMR spectroscopy [18,19]. The study showed weak but significant systematic variations in the human fecal metabolome related to BMI, physical fitness, and blood plasma LP levels. The subjects of this study are healthy older adults, include 83 males (70+/−5 age) and 80 females (70+/−5 age), recruited in the Counteracting Age-related Loss of Skeletal Muscle Mass (CALM) study [20]. The CALM study was designed to investigate the effect of increased protein supplementation and physical exercise on the general health and well-being of community-dwelling relatively healthy older Danes. The present paper scrutinized the fecal metabolome data obtained on all subjects at the baseline, pre-intervention time. Apart from physical fitness, the study showed fecal metabolic fluctuations according to the health-indicating parameters including BMI and plasma LP levels. The distributions of these parameters, including upper and lower limits of specific clinical parameters determined by the Danish Health Care system [21], are illustrated in Figure 1. Physical fitness in the CALM study was determined as a function of three variables, including (1) chair-rise test, (2) body composition measured with dexa-scan, and (3) BMI [14]. Using this strategy, all 163 CALM subjects at baseline were divided into two groups: low fitness (LF) and high fitness (HF).

Previous studies have reported that both BMI and physical fitness are closely related to the levels of blood LP [22]. Rashid et al. reported that physical exercise is an effective way for raising the HDL*chol* level [23]. Van Gaal et al. showed that BMI in premenopausal women is negatively correlated to the plasma levels of HDL*chol* and positively correlated to LDL*chol* [24]. Cuesta-Zuluaga et al. found a close relationship between gut microbiome dysbiosis, obesity, CVD risk factors, and fecal short-chain fatty acids (SCFAs) levels in community-dwelling adults [25]. However, the relationships between the human fecal metabolome, BMI, physical fitness, and CVD risk factors such as LPs are not well characterized, and the present study aimed to improve this knowledge gap.

## 2. Results

### 2.1. Human Fecal Metabolome of Older Adults

The human fecal metabolome was investigated by employing two analytical platforms, namely ^1^H NMR spectroscopy and GC-MS. Absolute concentrations of 36 human fecal metabolites and the relative concentrations of 76 unknown spin systems were quantified from ^1^H NMR spectra, as described previously [16]. The mean absolute concentrations of the 36 human fecal metabolites are reported in Cui et al. [26]. These fecal metabolites covered five SCFAs, 13 AAs, five organic acids (OAs), two sugars, namely glucose and galactose, one nucleobase (uracil), and 11 other common metabolites, including ethanol, trimethylamine, methanol, carnitine, glycerol, guanidinoacetic acid, betaine, xanthine, acetone, and formic acid (Table S1, Cui et al., 2021). SCFAs and AAs were found to have the highest concentration in fecal metabolites in all subjects, regardless of sex, age, and fitness. Among the SFCAs, acetic acid was found to have the highest concentration, followed by propionic acid. Isoleucine was found to have the highest concentration among the AAs.

The GC-MS determined fecal metabolome included 149 peaks. Of these, 138 were identified at level 2 or 3 according to the Metabolomics Standards Initiative [27] using electron ionization–mass spectrometry (EI-MS) and retention index (RI)-based matching. A total of 49 metabolites were identified at level 2 (EI-MS match of >80% and ∆RI of <50), whereas 89 metabolites were identified at level 3, to a metabolite class level or to a metabolite candidate level with limited confidence (EI-MS match of >65%). Fecal metabolites detected by GC-MS represented six molecular classes, including 14 AAs, 34 fatty acids (FAs), 19 sugars, 30 OAs, 11 sugar alcohols, and 47 other metabolites, including esters, uridine, and glucosamine (Table S1). In addition, metabolites such as derivatives of SCFAs, including 2,2-dimethyl-3-hydroxybutanoic acid and 2-oxo-propanoate, and OAs such as benzeneacetic acid, mercaptoacetic acid, and gluconic acid, and a few other level 3-identified metabolites in Table S1 have not been previously reported in human fecal matter and require further validation in new independent studies.

### 2.2. Associations between the Human Fecal Metabolome and Physical Fitness

**Figure 2:**
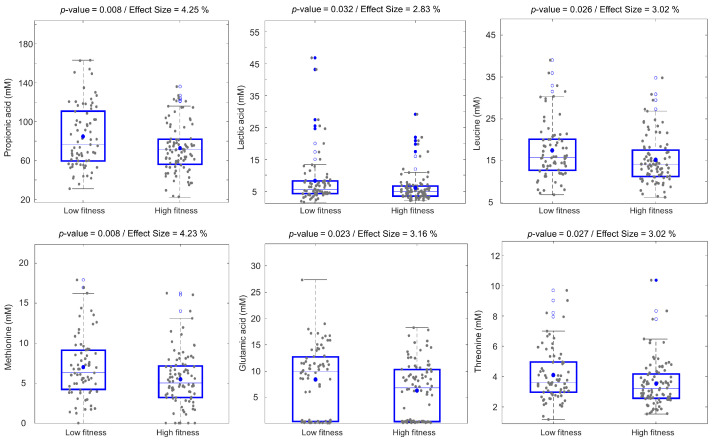
Boxplots of selected human fecal metabolites detected using ^1^H NMR spectroscopy that are found to be different between low- and high-fitness subjects (low fitness, *n* = 73; high fitness, *n* = 90). *p*-values and effect sizes (%) are calculated from ANOVA. The horizontal lines and dots inside the boxes represent median and mean values, respectively. Blue circles and blue-filled circles correspond to quartiles >75% and 95%, respectively.

In a previous study, physical fitness was defined from three variables, including the chair-rise test, body composition measured with dexa-scan and BMI [14]. In this study, the 163 older adults were divided into two groups according to physical fitness, 73 LF and 90 HF. In order to explore the effects of physical fitness, both univariate and multivariate analyses were carried out on the human fecal metabolome datasets generated using NMR and GC-MS. Multivariate data analysis methods, PCA and PLS-DA, revealed no clear differences in the fecal metabolomes of LF and HF groups. However, univariate analysis by means of one-way analysis of variance (ANOVA) revealed that 11 and 17 metabolites, quantified from NMR and GC-MS, respectively, were significantly different between LF and HF participants (Table S2). These metabolites included AAs such as leucine, isoleucine, valine, alanine, methionine, glutamic acid, sarcosine, threonine, phenylalanine, glycine, and other metabolites such as propionic acid, oxalic acid, nicotinic acid, lactic acid, cyclohexanecarboxylic acid, xylopyranose, and pentanol. Box plots of the six most significant metabolites, including leucine, methionine, glutamic acid, threonine, propionic acid, and lactic acid, are shown in Figure 2. All these metabolites showed a slight increase in their mean concentrations in the fecal samples of LF subjects. Propionic acid and methionine were the two most significant metabolites, displaying the greatest effect size (>4%). In addition, one-way ANOVA was applied to test the significance of the fitness effect in males and females separately (Table S3). The results indicated that methionine and threonine had significantly higher concentrations in the LF males compared to HF males, while fecal levels of propionic acid, malic acid, and fumaric acid were higher in LF females compared to HF females. A total of 13 metabolites detected using GC-MS, including ethenyl acetate, oxalic acid, 2-hydroxyisovaleric acid, and arabinopyranose, showed significantly increased concentrations in HF males compared to LF males. Seven metabolites, such as carbodiimide, oxalic acid, glutaric acid, and xylopyranose, were elevated in LF females compared to HF females. Ethenyl acetate (*p*-value = 0.006, effect size = 10.6%) and oxalic acid (*p*-value = 0.022, effect size = 7.7%) were the two metabolites with the largest effect size related to the fitness effect when studying males and females separately, and they were higher in HF males and females compared to the LF groups.

### 2.3. Fecal Metabolic Differences between Overweight/Obese and Normal BMI Older Adults

**Figure 3:**
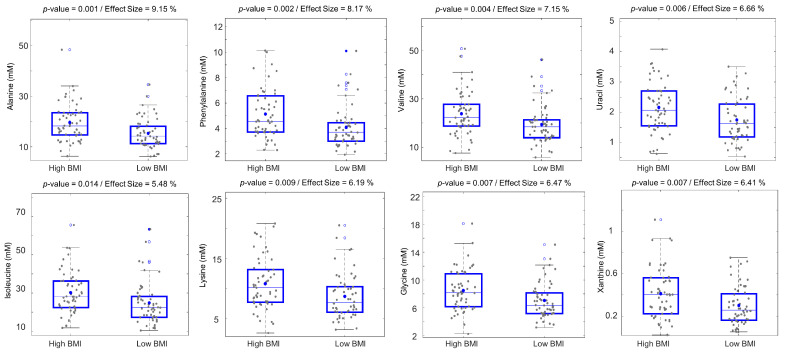
Boxplots of selected human fecal metabolites that are different between low-BMI (<23.5, *n* = 54) and high-BMI (>27, *n* = 55) groups. *p*-values and effect sizes (%) were calculated from ANOVA. The horizontal lines and dots inside the boxes represent median and mean values, respectively. Blue circles and blue-filled circles correspond to quartiles >75% and 95%, respectively.

BMI is an important anthropometric factor reflecting human health and well-being [28]. Although BMI was one of the factors included for dividing older adults into the low- and high-physical-fitness groups, investigating the effect of BMI alone on the individual human fecal metabolic differences is of interest to understand the differences in the function of human gut microbiota in relatively overweight/obese and normal-BMI older adults. Therefore, all subjects were sorted according to BMI, and the bottom 1/3 tertile (BMI < 23.5, *n* = 54) and the top 1/3 tertile (BMI > 27, *n* = 55) subjects were classified as low-BMI and high-BMI groups, respectively. As in the case of physical fitness, the human fecal metabolomics data were then evaluated with respect to BMI groups using univariate and multivariate data analysis methods. No systematic differences were found between the fecal metabolic profiles of low-BMI and high-BMI subjects using multivariate data analysis. In contrast, one-way ANOVA identified 14 metabolites from the ^1^H NMR analysis that were significantly different between the two BMI groups (Table S4). These metabolites were AAs (leucine, valine, alanine, lysine, glycine, phenylalanine, methionine, tyrosine, glutamic acid, and isoleucine), SCFAs (propionic acid and pyruvic acid), uracil, and xanthine (Figure 3). All these metabolites displayed higher average concentrations in the high-BMI group. This trend remained constant when analyzing males and females separately (Table S5). In addition, 12 metabolites displayed significantly increased concentrations in high-BMI males compared to low-BMI males, and the majority of these were also found to be higher in high-BMI subjects when evaluating both sexes together (Table S4). Three metabolites, glutamic acid, malic acid, and fumaric acid, were found to be higher in the high-BMI-females group compared to the low-BMI-females group.

### 2.4. Associations between Blood Lipoproteins and the Fecal Metabolome

**Figure 4:**
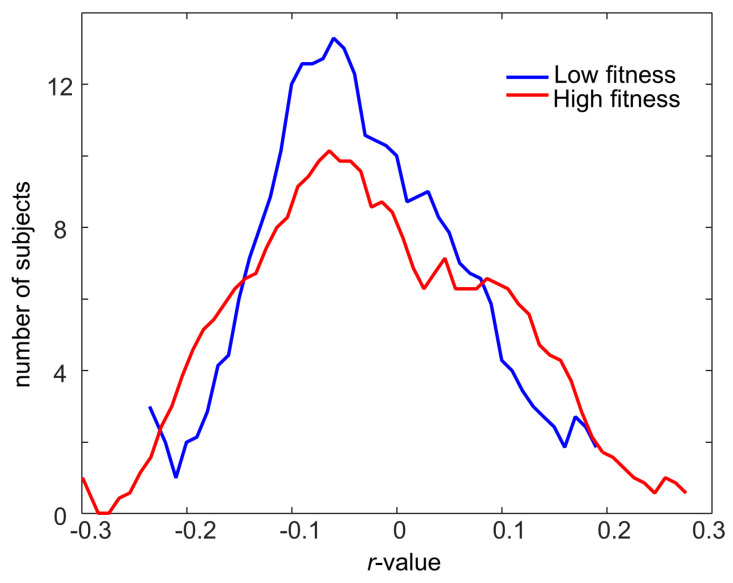
Distribution of Pearson’s correlation coefficients (*r*-values) calculated between 36 fecal metabolites including valeric acid, isovaleric acid, leucine, isoleucine, valine, propionic acid, ethanol, lactic acid, alanine, butyric acid, acetic acid, methionine, acetone, glutamic acid, pyruvic acid, aspartic acid, sarcosine, trimethylamine, lysine, malonic acid, methanol, carnitine, glycine, glycerol, guanidinoacetic acid, betaine, threonine, malic acid, glucose, galactose, uracil, fumaric acid, tyrosine, phenylalanine, xanthine, and formic acid and eight lipoproteins including total *chol*, HDL*chol*, LDL *chol*, VLDL*chol*, total *chol*/HDL*chol*, total *chol*/HDL-Apo A, LDL*chol*/HDL*chol* and VLDL*chol*/HDL*chol*. The correlation analysis was performed separately for low fitness (blue) and high fitness subjects (red).

The relationship between human blood plasma LP and the fecal metabolome is largely limited to studies investigating fecal cholesterol excretion [29], while untargeted fecal metabolomics has, to our knowledge, not been explored in the context of LP distribution in blood. Accordingly, human fecal metabolic differences in older adults were explored according to their plasma levels of cholesterol (*chol*), HDL*chol*, LDL*chol*, very-low-density lipoproteins particles (VLDL*chol*), and HDL-Apolipoprotein A (Apo A), and their ratios. First, using all subjects, Pearson’s correlation coefficients were calculated between all human fecal metabolites and plasma LPs, including total *chol*, HDL*chol*, LDL*chol*, VLDL*chol*, and *chol* ratios, such as total *chol*/HDL*chol*, total *chol*/HDL-Apo A, LDL*chol*/HDL*chol*, and VLDL*chol*/HDL*chol*, to evaluate any possible correlation (Table S6). The results indicated that the level of total *chol* displayed a weak negative correlation with the fecal concentrations of lactic acid (*r*-value = −0.2, *p*-value = 0.006), ethanol (*r*-value = −0.19, *p*-value = 0.02), and formic acid (*r*-value = −0.19, *p*-value = 0.02). The ratio of total *chol* to HDL-Apo A also showed weak negative correlations with the fecal concentrations of isoleucine (*r*-value = −0.17, *p*-value = 0.03), methionine (*r*-value = −0.17, *p*-value = 0.03), threonine (*r*-value = −0.18, *p*-value = 0.024), uracil (*r*-value = −0.18, *p*-value = 0.02), and phenylalanine (*r*-value = −0.16, *p*-value = 0.04). The same correlation analysis was performed on sex or BMI-stratified subgroups. The distribution plots (Figure S1) represent an overview of the *r*-values calculated from the correlations between fecal metabolite levels of males, females, low-BMI, and high-BMI groups with the eight different selected fractions of cholesterols. The results showed that all *r*-values of the six groups were distributed in the range of −0.4 to 0.4. The distribution plots of Figure 4 and heatmaps of Figure S2 represent the Pearson’s correlation coefficients calculated between eight LP variables and 36 fecal metabolites in the six different subpopulations, including LF, HF, males, females, low BMI, and high BMI. The results indicated that correlations of fecal metabolites with plasma LPs were different when subjects were stratified according to fitness, sex, and BMI. Fecal metabolites of HF subjects showed more positive correlations with HDL*chol* level than in LF subjects, and similarly, HF subjects displayed more negative correlations with total *chol*. Fecal metabolites in males displayed more negative correlations with levels of total *chol*, LDL*chol*, and total *chol*/HDL-Apo A than in females. Low-BMI subjects showed greater positive correlations between their fecal levels of valeric acid and glucose with plasma total *chol*, as well as between the level of carnitine and HDL*chol*, when compared to high-BMI subjects, while high-BMI subjects showed significant negative correlations between fecal levels of threonine, betaine, and isoleucine and the total *chol*/HDL-Apo A ratio. Similarly, plasma levels of HDL*chol* were found to be at higher levels in HF subjects, whilst LF subjects displayed higher concentrations of LDL*chol* and IDL*chol* cholesterol fractions (Trimigno et al., in press).

In order to further investigate a possible relationship between the human fecal metabolome and plasma LP, subjects were sorted according to their plasma concentrations of selected LPs or their ratio, some of which are known to be a risk factor for CVD, and classified either as low-LP (bottom 1/3) or high-LP (top 1/3) subjects (Table S7). Then, the fecal metabolome datasets, both from NMR and GC-MS, were scrutinized to reveal fecal metabolic differences between the low-LP and high-LP subjects. One-way ANOVA identified 16 fecal metabolites being different between the low- and high-LP groups (Table 1). These metabolites included two SCFAs (butyric acid and isovaleric acid), 11 AAs (valine, leucine, isoleucine, alanine, glutamic acid, proline, aspartic acid, lysine, glycine, methionine, and phenylalanine), methanol, lactic acid, and uracil. Almost all the metabolites displayed a higher concentration in the low-LP group, except for the two SCFAs, butyric acid and isovaleric acid. Butyric acid showed an increased concentration in the high-HDL*chol* group, while isovaleric acid showed an increased concentration in the high-VLDL*chol* group. In addition, the ratios between LP variables, which have been suggested as CVD risk factors [30], such as total *chol*/HDL*chol,* total *chol*/HDL-Apo A, LDL*chol*/HDL*chol*, and VLDL*chol*/HDL*chol*, were found to be related to the fecal levels of SCFAs, AAs, methanol, and uracil. The level of butyric acid showed increased concentrations in low-LDL*chol*/HDL*chol* and VLDL*chol*/HDL*chol* groups compared to the corresponding high-LP groups. Amino acids such as leucine, isoleucine, valine, phenylalanine, glycine, lysine, methionine, lactic acid, and uracil had higher concentrations in the low-total-*chol*/HDL-Apo A group compared to the high-LP-ratio group. In addition, fecal concentrations of valine, alanine, glutamic acid, and methanol were found to be higher in subjects with a low total *chol*/HDL*chol* ratio.

## 3. Discussion

One of the key steps to improve the quality of life and well-being of older adults is to better understand factors influencing their physical fitness. In the CALM study, almost half of the participants were either overweight or obese with high total blood cholesterol (>5.7 mmol/L) levels, which is considered a CVD risk factor. High BMI and total *chol* in older adults can be explained by low basal metabolism, dietary habits, and lifestyle. On the other hand, only a few of the participants were on the borderline or within the actual risk zone in terms of plasma levels of total *tg*, VLDL*chol*, LDL*chol*, HDL*chol*, and total *chol*/HDL*chol*. More than 90% of the participants were found to be in the normal range in terms of concentrations of the above LP. The roles of gut health and alterations in the fecal metabolome in chronic diseases have been characterized in a few studies, while their relations to physical fitness are still poorly understood. A previous study showed that older adults differing in their physical fitness also have differences in their gut microbiome and glucose metabolism [14].

The current study shows how the fecal metabolome differs between the two fitness groups (LF and HF). The differences were weak but significant and covered a wide range of metabolites, such as SCFAs and AAs, which are important molecules for the maintenance of a healthy gut microbiome. AAs and SCFAs are functional metabolites regulated by the gut microbiome, and SCFAs are generated by bacterial fermentation that is specific to dietary fibers and resistant starch in the colon [31]. They are also found to be the metabolites that differ the most between LF and HF groups. An increasing number of studies have provided evidence that SCFAs also exert important physiological effects on human health [32,33]. Ortiz-Alvarez et al. reported that fitness is playing a key role in the secretion of SCFAs, with increased fecal SCFA concentrations found in a long-term exercise intervention group [34]. Barton et al. found that acetic acid, propionic acid, and butyric acid concentrations are increased in the gut of trained subjects (male rugby players) [35]. However, in this study, we can only confirm that the fecal levels of propionic acid and lactic acid were slightly increased in LF subjects compared to HF subjects. This discrepancy may be because the study by Barton et al. was performed as a long-term fitness intervention, while the present study only included baseline fitness levels of free-living community-dwelling older adults who have not (yet) been under specific physical training. Moreover, the older subjects in this study showed significantly lower concentrations of SCFAs than younger, 18-years-old, subjects did, as previously reported [26]. However, the increased concentrations of SCFAs in LF subjects may also be explained by the altered regulation of glucose transport, fatty acid oxidation, and steroid biosynthesis in LF subjects compared to HF subjects [36,37], and this increase has also been related to different rates of glucose homeostasis and lipid metabolism between HF and LF subjects [38].

Fecal AAs were also found at increased concentrations in LF subjects. AAs are the building blocks of proteins and enzymes, and they are partially digested and absorbed in the intestine. It has been shown that LF subjects were eating more protein than the HF subjects [14]. Although this difference in protein consumption does not influence the fecal AA levels, it weakly affects the isovaleric acid and propionic acid levels [26]. In feces, AAs derive from the endogenous metabolism of the gut microbiome [39], and the effect of fitness on changing fecal AA levels is elusive [40]. Deda et al. [39] reported similar results regarding AA changes in feces, with lower levels found after high-exercise training. These differences in AA levels have been suggested to be linked with diet composition, which, in turn, generates the growth of gut microbes such as *Firmicutes*, which are crucial for AA and carbohydrate metabolism [41]. In addition, physical fitness has also been related to a higher fecal alpha bacteria diversity, further influencing the AA and SCFA levels [34]. Similar to our findings, Kujala et al. showed a lower level of isoleucine in the serum of the higher-fitness subjects compared to lower-fitness subjects. Sakaguchi et al. found that the concentrations of AAs, including leucine, isoleucine, alanine, methionine, lysine, and glutamic acid, in human plasma increase after 14 h of heavy exercise [42].

Ethenyl acetate and oxalic acid that are increased in the feces of HF males compared to LF males are potentially harmful to human health. For example, ethenyl acetate is considered a possible carcinogenic compound [43], and oxalic acid is considered to be a natural antioxidant in some systems [44], while increased levels of AAs in LF subjects compared to HF subjects might be related to the malabsorption in older adults [45].

High BMI is considered a risk factor for T2D, and lowering BMI is important for regulating T2D [46]. In this study, we found 14 fecal metabolites significantly different between low-BMI (<23.5) and high-BMI (>27) subjects. Nogacka et al. found that the number of *Bacteroidetes* was significantly downregulated in overweight or obese subjects, which may be related to the BMI effects observed in this study [47]. A study by Liang et al. demonstrated that the human fecal metabolome composition can be associated with diabetes, with fecal homocysteine and butanone levels being significantly increased in diabetic patients compared with controls, whereas the levels of L-malic acid and dibutyl decanedioate were decreased in diabetic patients [48]. In agreement with our results, Liang et al. concluded that AA levels in feces are greater in subjects who are at high risk of diabetes compared to lower-risk subjects. In addition, Zhou et al. showed that the fecal level of phenylalanine is higher in feces of T2D patients compared to patients treated against T2D [49]. The elevated levels of certain AAs in older adults with high BMI are hypothesized to be due to impaired metabolism and specific gut microbiota promoted in overweight or obese subjects. The high-BMI subjects are expected to have lower gut microbiota diversity, which may generate specific effects on gut metabolites. Combined, these findings provide evidence that the fecal metabolome can give an indication of impaired metabolism due to obesity, which are primarily pronounced as increased levels of fecal AAs such as valine, alanine, phenylalanine, and tyrosine.

Human blood plasma lipoproteins have long been studied as a ‘risk factor’ for CVD and liver diseases [50]. The analysis of the CALM cohort showed that high BMI, thus low-fitness, subjects have a decreased level of plasma HDL*chol*, increased level of LDL*chol*, and intermediate-density lipoprotein cholesterol (IDL*chol*) (Trimigno et al., in press). Similar to the findings of Trimigno et al., Pasiakos et al. reported that lower-BMI subjects have higher HDL*chol* levels at high-protein diets [51], which indicates that high-BMI subjects potentially have a higher risk of CVD as HDL*chol* is recognized as good cholesterol [52]. The higher risk of CVD for overweight subjects has also been related to impaired HDL*chol* metabolism [53]. Schmedes et al. concluded that weight loss decreases both the levels of LP in plasma and SCFA in fecal samples [54].

Therefore, the correlations found between fecal metabolites and certain LP levels indicate that research in this direction may provide new insights into a link between human metabolism/health and the gut microbiome. In this paper, a comparison of the fecal metabolite concentrations of two groups (low LP and high LP) was performed. The results indicated that a normal (according to the Danish health care system) concentration range of plasma HDL*chol* level is associated with an increased fecal butyric acid level compared to those older adults whose plasma HDL*chol* level was lowered (<1.1 mmol/L). Subjects with lower HDL*chol* levels instead showed higher fecal levels of isovaleric acid, suggesting that an increase of this SCFA in the fecal matter may be a sign of a higher risk of CVD. Similarly, Zheng et al. found that the blood lipid and LP levels were negatively correlated with the blood metabolome, SCFAs including acetic acid, butyric acid, and propionic acid in feces were related to blood cholesterol levels in overweight women [3]. Diet may be a key factor explaining the links between LP levels and fecal SCFA as dietary fat and carbohydrate intake have a significant effect on fecal SCFA levels in humans [26]. These findings on LP and the fecal metabolome can potentially provide useful information for the design of a diet that could reduce the risk of CVD in hyperlipidemia patients. In addition, we have documented that LDL*chol*, *tg*, and cholesterol ratios, including (total *chol*/HDL*chol*) and total *chol*/HDL-Apo A, are related (more sensitive) to the fecal levels of AAs in older adults. This is an interesting finding as AAs are also important building blocks of proteins and, thus, may be key for determining their functions. Our study also showed that increased fecal levels of AAs, including leucine, isoleucine, valine, phenylalanine, glycine, lysine, and methionine, were related to a normal (healthy) concentration range of good cholesterol (HDL*chol*), and thus may be a sign of low risk of CVD. The changes in fecal AA levels may have a close relationship with the transport and reduction of lipids in older adults [55], but may also be linked to diet [26]. Especially, dietary intake of fat might be one of the reasons (confounders) explaining the increased fecal isoleucine and valine levels in high BMI subjects. These specific AAs are likely the most sensitive fecal metabolites reflecting BMI or the levels of plasma LP in humans. Understanding the mechanisms, direct or indirect relationships in between the changes in human fecal metabolome with BMI or LP concentrations, is particularly important to adjust the dietary and lifestyle recommendations for older adults to reduce the risk of CVD. Future studies are required to validate correlations found in this study between the human fecal metabolome and physical fitness, BMI, and plasma LP levels, which will open new perspectives for better understanding the role of the human gut microbiome in the health and well-being of older adults.

## 4. Materials and Methods

### 4.1. Sample Collection

All subjects included in this study were apparently healthy older adults, community-dwelling, and self-supportive. Participants were recruited through local newspapers, magazines, radio programs, social media, and presentations at senior centers and public events [56]. Anthropometric, body-composition, and physical performance measurements, average daily physical activity, dietary intake and preferences, microbiome composition, and clinical biomarkers were recorded for each participant, and fecal samples were collected [14]. A total of 163 subjects (83 males and 80 females) who provided a sufficient amount of fecal samples were included in this study out of 205 subjects recruited in the CALM project. All subjects who participated in the CALM project also provided written informed consent. Procedures of the CALM project (Clinical Trials NCT02115698) were approved by the Danish Regional Ethical Committees of the Capital Region (J-nr. H-4-2013-070) and performed according to the Declaration of Helsinki II [57]. After collection, fecal samples were immediately stored in the fridge at 4 °C for a maximum of 48 h and subsequently stored at −80 °C until NMR analysis. The metadata, including sex, age, height (cm), body weight (kg), and individual dietary energy and macronutrient intake, were collected for each participant. Blood samples were collected for measuring VLDL*chol*, LDL*chol*, HDL*chol*, total tg, total *chol*, and their subclasses. More details of the metadata collected in the CALM project have been previously reported [14,56].

### 4.2. Chemicals

Chemicals and reagents used in this study were purchased from Sigma-Aldrich (Søborg, Denmark). The chemicals included deuterium oxide (D_2_O, 99.9 atom % D), monobasic potassium phosphate (KH_2_PO_4_, ≥99.0%), dibasic potassium phosphate (K_2_HPO_4_, ≥98.0%), sodium salt of 3-(trimethylsilyl) propionic-2,2,3,3-d4 acid (TSP, 98 atom % D, ≥98.0%), sodium chloride (NaCl, ≥99.0%), methanol (CH_3_OH, ≥99.98%, containing 10 ppm of palmitic-acid methyl ester and 10 ppm of sorbitol as internal standards), and sodium azide (NaN_3_, ≥99.5%). The water used throughout the study was purified using a Millipore lab water system (Merck KGaA, Darmstadt, Germany) equipped with a 0.22 μm filter membrane.

### 4.3. Sample Preparation for Metabolomics Analysis

#### 4.3.1. Fecal Sample Preparation for ^1^H NMR Metabolomics Analysis

Fecal samples were thawed at 4 °C and mixed with ultrapure water at a *w*/*v* ratio of 1:2 (feces/water) and transferred into filter bags for homogenizing for 1 min (Lab Seward, BA7021, Seward, AK, USA); the fecal slurry was then stored at −80 °C before further analysis. Before the metabolite extraction for NMR analysis, fecal slurry samples were thawed at 4 °C, and 600 µL of the slurry was transferred into a 1.5 mL plastic Eppendorf tube and then mixed with 600 µL of phosphate buffer, followed by vortexing for 2 min. The mixture was then centrifuged at 6 °C for 30 min at 14,000× *g*. Finally, 600 µL of clear supernatant was transferred into a 5 mm O.D. SampleJet NMR tube for ^1^H NMR analysis. At least one control sample was included in the NMR analysis between 10 real samples. Phosphate buffer was employed as the extraction solvent; details of the fecal sample preparation protocol and phosphate buffer preparation have been described in Cui et al. [16].

#### 4.3.2. Fecal Sample Preparation for GC-MS Metabolomics Analysis

A volume of 1 mL of the homogenized fecal flurry (as described earlier) was mixed with 1 mL of phosphate buffer, and then quickly frozen in liquid nitrogen and freeze-dried overnight. Twenty milligrams of the freeze-dried fecal powder sample was then re-suspended in 1 mL of methanol, vortexed, and centrifuged for 30 min at 12,000× *g* at 4 °C. Fifty microliters of the supernatant was then dried using a ScanVac (Labogene, Lynge, Denmark) at 1000 rpm for 3 h at 40 °C. Immediately after drying, samples were sealed with airtight magnetic lids into 2.0 mL GC-MS vials and derivatized in two steps using a Dual-Rail MultiPurpose Sampler (MPS) (Gerstel, Mülheim an der Ruhr, Germany): (i) addition of 10 µL of MEOX reagent (20 mg mL^−1^ of methoxiamine hydrochloride in dry pyridine) followed by agitation at 45 °C for 90 min by mixing at 750 rpm, (ii) addition of 40 µL of TMS reagent, trimethylsilyl cyanide (TMSCN) [58], followed by agitation at 45 °C for 45 min by mixing at 750 rpm. All steps involving sample derivatization and injection were automated using the MPS, which was equipped with a sample agitation unit. Immediately after derivatization, 1 μL of the derivatized sample was injected into a cooled injection system (CIS4) (Gerstel, Mülheim an der Ruhr, Germany) port in splitless mode. More details of the targeted and untargeted GC-MS fecal sample preparation protocol and phosphate buffer preparation have been described by Castro-Mejía et al. [14].

### 4.4. Data Acquisition

#### 4.4.1. ^1^H NMR Spectroscopy Analysis

^1^H NMR spectra were recorded at the University of Copenhagen (Department of Food Science) using a Bruker Advance III 600 MHz NMR spectrometer equipped with a 5 mm broadband inverse RT (BBI) probe, automated tuning and matching accessory (ATMA) and cooling unit BCU-05, and an automated sample changer (Sample Jet, Bruker Biospin, Rheinstetten, Germany) with sample cooling (278 K) and preheating stations (298 K). Data acquisition and processing were carried out using TOPSPIN 3.5 PL6 (Bruker Biospin, Rheinstetten, Germany). The Icon NMR (Bruker Biospin, Rheinstetten, Germany) interface was used to control the automation of the overall measurement procedure. Automatic tuning and matching, lock, and shimming were performed using TOPSPIN 3.5 PL6. The standard pulse sequence with water suppression (*noesygppr1d*) was employed for measuring the one-dimensional ^1^H NMR spectra. A total of 32 scans were acquired after 4 dummy scans, and the generated free induction decays (FIDs) were collected into 128 k data points using a spectral width of 20 ppm. Acquisition time, recycle delay, and mixing time were set to 2.72, 4.0, and 0.01 s, respectively. The receiver gain was set to 90.5. A more detailed description of NMR analysis has been reported in a previous paper [16].

#### 4.4.2. GC-MS Data Acquisition

The GC-MS setup was made by combining an Agilent 7890B gas chromatograph (GC) (Agilent Technologies, Santa Clara, CA, USA) with a time-of-flight mass spectrometer, HT Pegasus TOF-MS, (LECO Corporation, Saint Joseph, MO, USA). A Zebron ZB 5% Phenyl 95% dimethylpolysiloxane column (30 m with I.D. 250 μm and film thickness 0.25 μm) with a 5 m inactive guard column (Phenomenex, Torrance, CA, USA) was employed for GC separation. A hydrogen generator, Precision Hydrogen Trace 500 (Peak Scientific Instruments Ltd., Inchinnan, UK), was used to supply a carrier gas at a constant column flow rate of 1.0 mL min^−1^. The initial temperature of the GC oven was set to 40 °C, and this temperature was held for 2 min, followed by heating at the rate of 10 °C min^−1^ to reach 320 °C, and then held for 5 min at 320 °C. The mass spectra were recorded at the mass-to-charge (*m/z*) range of 45–600, and the scanning frequency was set to 10 scans s^−1^. The MS detector and ion source were switched off during the first 6.3 min of solvent delay time, and the transfer line and ion source temperature were set to 280 °C and 250 °C, respectively. The mass spectrometer was tuned according to the manufacturer’s recommendation using perfluorotributylamine (PFTBA). MPS and GC-MS were controlled using vendor software Maestro (Gerstel, Mülheim an der Ruhr, Germany) and ChromaTOF (LECO Corporation, Saint Joseph, MO, USA), respectively. A more detailed description of GC-MS analysis has been reported in a previous paper [14].

### 4.5. Processing of the Raw ^1^H NMR Spectra and GC-MS Data

The acquired ^1^H NMR spectra were processed using SigMa [59]. First, they were normalized to the Electronic Reference To access In vivo Concentrations (ERETIC) signal [60]. Then, reference alignment to the TSP signal was carried out, followed by pre-alignment of 98 spectral regions. 83 signature signals (SS) and 76 signals of unknown spin-system (SUS), were then identified from the fecal NMR spectra, aligned, and quantified using the SigMa software as described in the previous study [16]. In addition, 49 BINs (intervals of complex regions representing more than one metabolite) were identified and quantified by summing all data points. Absolute concentrations (ACs) of 36 metabolites were calculated in SigMa using the areas of the corresponding SS, a corresponding proportion of a given proton in a metabolite, and the area of the ERETIC signal, which corresponds to 10 mM of protons. Details of the quantification and distributions of metabolites’ concentrations can be found in the study by Cui et al. [26].

The raw GC-MS data were processed using the Statistical Compare toolbox of the ChromaTOF software (Version 4.50.8.0, LECO Corporation, Saint Joseph, USA). Deconvoluted mass spectra were used for peak identification using LECO-Fiehn and NIST11 libraries. The library search was set to return the top 10 hits with an EI-MS match of >75% using the normal-forward search and with a mass threshold of 20. Deconvoluted peaks were aligned across all samples using the following settings: retention time shift allowance of <3 s, EI-MS match of >95%, mass threshold of >25, and present in >90% of all pooled control samples. The details of metabolite identification from GC-MS data have been described in a previous paper [61].

### 4.6. Statistical Analysis

Multivariate analyses, including principal component analysis (PCA) [62], analysis of variance-simultaneous component analysis (ASCA) [63], and partial least squares (PLS) regression [64], as well as one-way ANOVA with Benjamini–Hochberg’s multiple test correction, and the FDR (False Discovery Rate) rate of 10% [65], were performed on NMR and GC-MS metabolite data to find possible effects of physical fitness, BMI, and LP on the human fecal metabolome. All significant metabolites have also been evaluated using the Monte Carlo test (*N = 2000*). Metabolite data were checked for normal distribution prior to ANOVA. All statistical analyses were performed on MATLAB (version R2015a, the Mathworks, Inc., Natick, MA, USA), and customized MATLAB scripts were written by the authors.

## 5. Conclusions

This study investigated the correlations between human fecal metabolome and physical fitness, BMI, and blood plasma concentrations of LP in healthy older adults. Overall, weak correlations were found between fitness and the fecal metabolome, with decreased concentrations of SCFAs (propionic acid and lactic acid) and AAs (methionine, leucine, glutamic acid, and threonine) in the high-fitness group. The effect of BMI on the concentrations of fecal metabolites is stronger, with increased concentrations of AAs (valine, alanine, and phenylalanine) and uracil in subjects with a high BMI. When the effect of BMI was investigated separately for low- and high-fitness subjects, main alterations in the fecal metabolome were pronounced on SCFAs and AAs, indicating a different function of the gut microbiome. The weak effects of LP levels and cholesterol ratios on the fecal metabolome were reflected on the levels of SCFAs (butyric acid and isovaleric acid) and AAs (valine, glutamic acid, phenylalanine, etc.). High LDL*chol* levels were related to decreased levels of valine, glutamic acid, phenylalanine, and lactic acid. We hypothesize that the diet composition, especially protein and fat intakes, is potentially a key factor determining the links between plasma LP levels and fecal SCFA levels, but more mechanistic-oriented studies are needed for validation. Thus, the results obtained in this study help to better understand how physical fitness and health biomarkers such as blood plasma LP are associated with the composition of the human fecal metabolome and, in turn, for linking these with gut microbiota function and human well-being.

## Figures and Tables

**Table 1:** A list of human fecal metabolites that have significantly different concentrations in older adults according to their blood plasma concentrations of selected lipoproteins. These lipoproteins included total *chol*, *tg*, HDL*chol*, LDL*chol*, VLDL*chol*, and the LP ratios of total *chol*/HDL*chol*, total *chol*/HDL-Apo A, LDL*chol*/HDL*chol*, and VLDL*chol*/HDL*chol*. MH = mean high LP, ML = mean low LP, total *chol* = total cholesterol, HDL*chol* = high-density lipoprotein cholesterol, LDL*chol* = low-density lipoprotein cholesterol, VLDL*chol* = very-low-density lipoprotein cholesterol, *tg* = triglyceride, HDL-Apo A = high-density lipoprotein-Apolipoprotein A, CVD = cardiovascular diseases.

Metabolite	CVD Risk Factor	*p*-Value	Effect Size (%)	Fold Change(MH/ML)
butyric acid	HDL*chol*	0.038	4.02	1.28
	LDL*chol*/HDL*chol*	0.033	4.20	0.78
	VLDL*chol*/HDL*chol*	0.044	3.73	0.79
isovaleric acid	HDL*chol*	0.001	11.08	0.80
	VLDL*chol*	0.013	5.63	1.29
leucine	LDL*chol*	0.030	4.55	0.85
	total *chol*/HDL-Apo A	0.016	5.28	0.84
	tg	0.039	4.63	0.76
valine	total *chol*/HDL*chol*	0.003	9.20	0.68
	total *chol*/HDL-Apo A	0.022	4.80	0.84
alanine	total *chol*/HDL*chol*	0.002	9.90	0.58
glutamic acid	total *chol*/HDL*chol*	0.020	5.02	0.67
	LDL*chol*	0.011	6.17	0.67
	LDL*chol*/HDL*chol*	0.025	4.68	0.68
phenylalanine	LDL*chol*	0.019	5.30	0.84
	total *chol*/HDL-Apo A	0.011	5.90	0.88
proline	tg	0.041	4.54	0.69
aspartic acid	tg	0.004	8.67	0.48
methanol	total *chol*/HDL*chol*	0.017	5.27	0.73
	LDL*chol*	0.020	5.22	0.66
glycine	total *chol*/HDL-Apo A	0.020	4.96	0.85
lysine	total *chol*/HDL-Apo A	0.040	3.87	0.85
methionine	total *chol*/HDL-Apo A	0.031	4.27	0.78
	LDL*chol*/HDL*chol*	0.038	4.01	0.80
lactic acid	total *chol*/HDL-Apo A	0.045	3.71	0.74
isoleucine	total *chol*/HDL-Apo A	0.023	4.74	0.84
uracil	total *chol*/HDL-Apo A	0.013	5.67	0.83

## Data Availability

The data presented in this paper is available on request from the corresponding authors.

## Supplementary Materials

The following are available online at https://www.mdpi.com/article/10.3390/metabo11110717/s1. Figure S1: Distribution of Pearson’s correlation coefficients *(r*-values) calculated between 36 fecal metabolites including valeric acid, isovaleric acid, leucine, isoleucine, valine, propionic acid, ethanol, lactic acid, alanine, butyric acid, acetic acid, methionine, acetone, glutamic acid, pyruvic acid, aspartic acid, sarcosine, trimethylamine, lysine, malonic acid, methanol, carnitine, glycine, glycerol, guanidinoacetic acid, betaine, threonine, malic acid, glucose, galactose, uracil, fumaric acid, tyrosine, phenylalanine, xanthine, and formic acid and eight lipoproteins including total *chol*, HDL*chol*, LDL*chol*, VLDL*chol*, total *chol*/HDL*chol*, total *chol*/HDL-Apo A, LDL*chol*/HDL*chol* and VLDL*chol*/HDL*chol*. The correlation analysis was performed separately for (A) males, (B) females, (C) low BMI and (D) high BMI groups. (BMI = body mass index), Figure S2: Heat-Maps made of Pearson’s correlation coefficients *(r*-values) calculated between 36 fecal metabolites including valeric acid, isovaleric acid, leucine, isoleucine, valine, propionic acid, ethanol, lactic acid, alanine, butyric acid, acetic acid, methionine, acetone, glutamic acid, pyruvic acid, aspartic acid, sarcosine, trimethylamine, lysine, malonic acid, methanol, carnitine, glycine, glycerol, guanidinoacetic acid, betaine, threonine, malic acid, glucose, galactose, uracil, fumaric acid, tyrosine, phenylalanine, xanthine, and formic acid and eight lipoproteins including total *chol*, HDL*chol*, LDL*chol*, VLDL*chol*, total *chol*/HDL*chol*, total *chol*/HDL-Apo A, LDL*chol*/HDL*chol* and VLDL*chol*/HDL*chol*. The correlation analysis was performed separately for (A) males, (B) females, (C) low BMI and (D) high BMI groups. (BMI = body mass index), Table S1: A list of fecal metabolites identified from the GC-MS data. Metabolites were identified by spectral matching using LECO-Fiehn and NIST11 libraries that are extracted from the NIST11 metabolite database. The table includes the metabolite names, metabolite class (class 1 = fatty acids, class 2 = amino acids, class 3 = organic acid, class 4 = sugars, class 5 = sugar alcohols, class 6 = other metabolites), occurrence (percentage frequency of detection of a metabolite across samples), calculated (in this study) and reported (in NIST11 metabolite library) retention indexes (RI), the difference between the reported RI and the calculated RI (ΔRI), identification level (Identified according to the Metabolomics Standards Initiative using electron ionization–mass spectrometry and retention index-based matching), EI-MS spectral match (R.Match), mean intensity(%) and the status of discovery of the metabolites in human fecal, Table S2: The results from the ANOVA analysis performed on NMR (A) and GC-MS (B) based metabolite data to investigate the differences between low fitness and high fitness groups including all subjects. (HF = high fitness, LF = low fitness), Table S3: The results from the ANOVA analysis performed on the sex stratified NMR and GC-MS based metabolite data to investigate the differences between low fitness (LF) and high fitness (HF) groups, Table S4: The results from the ANOVA analysis performed on NMR based metabolite data to investigate the differences between low BMI and high BMI groups including all subjects. MH = mean high BMI, ML = mean low BMI, Table S5: The results from the ANOVA analysis performed on the sex stratified NMR based metabolite data to investigate the differences between low BMI and high BMI groups, Table S6: The Pearson’s correlation coefficients and *p*-values calculated between the 36 fecal metabolites and the eight lipoproteins including total *chol*, HDL*chol*, LDL*chol*, VLDL*chol*, total *chol*/HDL*chol*, total *chol*/HDL-Apo A, LDL*chol*/HDL*chol* and VLDL*chol*/HDL*chol*, including all subjects. *Significant correlations, Table S7: The thresholds and numbers of subjects representing top 1/3 and bottom 1/3 according to the selected eight lipoproteins including total *chol*, HDL*chol*, LDL*chol*, VLDL*chol*, total *chol*/HDL*chol*, total *chol*/HDL-Apo A, LDL*chol*/HDL*chol* and VLDL*chol*/HDL*chol*.
