# Supplementary material for: Human Fecal Metabolome Reflects Differences in Body Mass Index, Physical Fitness, and Blood Lipoproteins in Healthy Older Adults
Source: Metabolites. 2021 Oct 21;11(11):717. doi: 10.3390/metabo11110717 (PMC8620003; doi:10.3390/metabo11110717)
Supplement: Supplementary file 1 [file metabolites-11-00717-s001.zip › Figure S1.pdf]

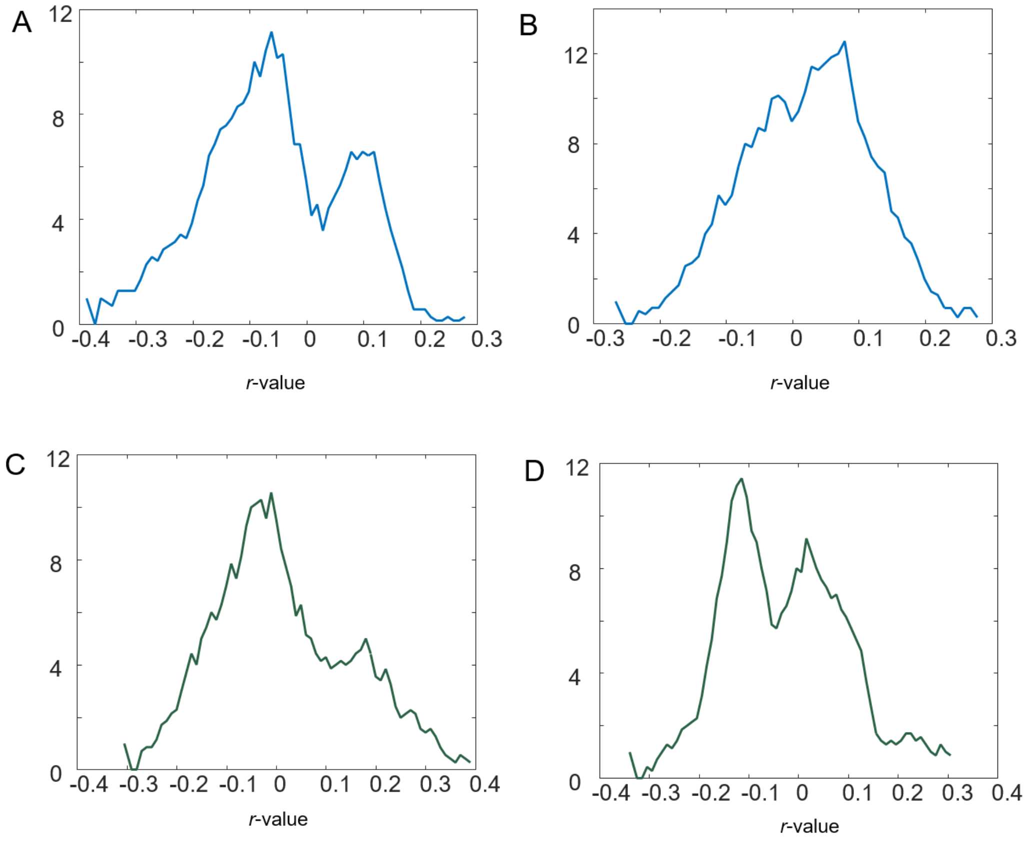

Figure S1. Distribution of Pearson's correlation coefficients (*r*-values) calculated between 36 fecal metabolites including valeric acid, isovaleric acid, leucine, isoleucine, valine, propionic acid, ethanol, lactic acid, alanine, butyric acid, acetic acid, methionine, acetone, glutamic acid, pyruvic acid, aspartic acid, sarcosine, trimethylamine, lysine, malonic acid, methanol, carnitine, glycine, glycerol, guanidinoacetic acid, betaine, threonine, malic acid, glucose, galactose, uracil, fumaric acid, tyrosine, phenylalanine, xanthine, and formic acid and eight lipoproteins including total *chol*, HDL*chol*, LDL*chol*, VLDL*chol*, total *chol*/HDL*chol*, total *chol*/HDL-Apo A, LDL*chol*/HDL*chol* and VLDL*chol*/HDL*chol*. The correlation analysis was performed separately for (A) males, (B) females, (C) low BMI and (D) high BMI groups. (BMI = body mass index).
