# Supplementary material for: Human Fecal Metabolome Reflects Differences in Body Mass Index, Physical Fitness, and Blood Lipoproteins in Healthy Older Adults
Source: Metabolites. 2021 Oct 21;11(11):717. doi: 10.3390/metabo11110717 (PMC8620003; doi:10.3390/metabo11110717)
Supplement: Supplementary file 1 [file metabolites-11-00717-s001.zip › Table S1.pdf]

Table S1. A list of fecal metabolites identified from the GC-MS data. Metabolites were identified by spectral matching using LECO-Fiehn and NIST11 libraries that are extracted from the NIST11 metabolite database. The table includes the metabolite names, metabolite class (class 1 = fatty acids, class 2 = amino acids, class 3 = organic acid, class 4 = sugars, class 5 = sugar alcohols, class 6 = other metabolites), occurrence (percentage frequency of detection of a metabolite across samples), calculated in (in this study) and reported (in NIST11 metabolite library) retention indexes (RI), the difference between the reported RI and the calculated RI (ΔRI), identification level (Identified according to the Metabolomics Standards Initiative using electron ionization–mass spectrometry and retention index-based matching), EI-MS spectral match (R.Match), mean intensity(%) and the status of discovery of the metabolites in human fecal.

No. Of Metabolites	Metabolite Names	Metabolite Classes	Occurrence	Retention Index (calculated)	Retention Index (reported)	Δ RI	Identification Level	R.Match	Mean (%) Intensity	Identified in fecal previously (1), never been reported in fecal before (0)
1	Isovaleric acid-TMS	1	30.22	952	932	20	2	912	0.7081	1
2	Sulfide-2TMS	6	96.64	969	993	24	2	849	0.9623	1
3	Carbodiimide-2TMS	6	93.66	974	650	324	3	806	1.0709	0
4	1,2-Ethenediol-2TMS	5	79.1	985	-	-	3	889	0.7882	1
5	Valeric acid-TMS	1	98.13	990	975	15	2	937	0.8493	1
6	Hexyl alcohol-TMS	6	23.96	1000	982	18	2	801	0.6870	0
7	Propylene glycol-2TMS	6	93.75	1009	1013	4	2	923	0.5908	1
8	1,3,5-Trioxepane	6	40.63	1022	771	251	3	887	0.8313	0
9	1,4,5,6-tetrahydropyridazine	6	86.94	1033	-	-	3	876	1.0094	1
10	Ethenoxyethane	6	95.90	1030	499	531	3	929	0.8383	0
11	Ethanolamine-2TMS	6	36.19	1034	1028.8	5.2	2	881	0.4678	1
12	Limonene	6	32.84	1040	1020	20	3	742	0.7390	1
13	Pentane-1,3,5-triol	5	47.01	1044	-	-	-	633	0.8060	0

14	Propane-1,3-diol-2TMS	5	34.33	1059	1073	14	3	715	0.6305	1
15	Lactic acid-2TMS	3	95.83	1064	1057	7	2	922	0.6227	1
16	Oxalic acid-2TMS	3	98.88	1066	1124	58	3	778	0.9615	1
17	Caproic acid-TMS	1	98.51	1076	1071	5	2	853	0.7118	1
18	Pyruvic acid-2TMS	1	86.45	1090	1100	10	2	825	0.7593	1
19	Oxalic acid-2TMS	3	95.83	1106	1124	18	2	789	0.9582	1
20	Amine-3TMS	6	99.25	1118	-	-	3	763	1.0453	1
21	Glycine-2TMS	2	95.15	1123	1105	18	2	918	0.7321	1
22	2-Hydroxybutyric acid-2TMS	3	80.21	1128	1126	2	2	821	0.5597	1
23	N-oxy-hydroxylamine-4TMS	6	91.66	1135	-	-	-	647	0.8202	0
24	Vinyl acetate	6	63.43	1156	564	592	3	935	0.5555	1
25	2-Methylphenol-TMS	6	64.58	1153	1127	26	2	863	0.6955	0
26	6-Azathiothymine	6	26.11	1158	-	-	3	693	0.4242	0
27	2-Hydroxyisovaleric acid-2TMS	1	82.29	1164	1178	14	2	814	0.6701	1
28	2-Butyl-1,3-dioxolane	6	32.09	1160	936	224	3	831	0.7215	0
29	N-Methyl-L-prolinol	5	76.04	1167	-	-	3	850	0.5275	1
30	Proline-2TMS	2	87.5	1175	1302	127	-	555	0.7699	1
31	β-Alanine-2TMS	2	16.41	1190	-	-	3	797	0.3432	1
32	Carbamate-3TMS	6	44.4	1196	-	-	3	824	0.7134	1
33	Cyclohexanecarboxylic acid-TMS	3	72.30	1201	1118	83	3	875	0.3837	1
34	2-Butanol	6	18.75	1214	-	-	3	907	0.7806	1
35	L-Valine-2TMS	2	100.00	1212	1234	22	2	875	0.9924	1

36	2-Hydroxy-3-methylvaleric acid-2TMS	1	71.88	1236	1253	17	3	751	0.5674	1
37	2-Hydroxyisocaproic acid-TMS	1	86.46	1233	1250	17	3	853	0.6512	1
38	Benzoic acid-TMS	3	99.25	1247	1232	15	2	947	0.7312	1
39	Diethylene glycol-2TMS	5	27.24	1242	1227	15	3	666	0.5861	1
40	N-[4-(Dimethylamino)butyl]-N-methylacetamide	6	81.34	1262	-	-	3	767	0.6369	0
41	Oxalic acid-2TMS	3	48.95	1266	1124	142	3	690	0.5195	1
42	L-Leucine-2TMS	2	93.75	1272	1272	0	2	803	0.9659	1
43	Nicotinic acid-TMS	3	81.34	1294	1274	20	2	840	0.8190	1
44	L-Leucine-2TMS	2	95.83	1294	1272	22	2	872	0.7555	1
45	L-Proline-2TMS	2	97.01	1295	1302	7	2	821	0.8812	1
46	Phenylacetic acid-TMS	3	100.00	1298	1273	25	2	820	0.8318	1
47	Succinic acid-2TMS	3	96.87	1313	1314	1	2	860	0.5977	1
48	Pyrimidine-2(O-TMS)	6	99.25	1335	1326	9	2	816	0.9555	1
49	Propanedioic acid-2TMS	3	88.54	1342	-	-	3	742	0.9600	1
50	Fumaric acid-2TMS	3	78.73	1350	1365	15	2	880	0.7821	1
51	5-Hydroxyhexanoic acid-2TMS	1	98.80	1354	1370	16	2	812	0.9708	1
52	2-(2-Methylbutyl)oxirane	6	30.20	1360	-	-	3	666	0.7843	0
53	2-Methyl-3-hydroxybutyric acid-2TMS	1	40.62	1375	1210	165	3	707	0.1618	1
54	Pyrimidine, 5-methyl-2(O-TMS)	6	97.38	1395	1400	5	3	679	0.9262	1

55	Aziridine-TMS	6	92.53	1339	-	-	3	728	0.8967	1
56	Glutaric acid-2TMS	3	92.16	1403	1400	3	2	796	0.7128	1
57	Hydrocinnamic acid-TMS	3	69.40	1415	1397	18	2	862	0.6369	1
58	2,2-Dimethyl-3-hydroxybutanoic acid-2TMS	1	29.40	1434	1395	39	3	812	0.3690	0
59	Decanoate-2TMS	6	97.70	1456	1455	1	2	895	0.7873	1
60	Malonic acid-TMS	3	21.80	1493	1190	303	3	670	0.3286	1
61	Malic acid-3TMS	3	44.70	1487	1390	97	3	743	0.5463	1
62	Adipic acid-2TMS	3	83.30	1504	1525	21	3	762	0.7921	1
63	L-Methionine-3TMS	2	53.35	1515	1530	15	3	686	0.5525	1
64	Proline-5-oxo-2TMS	2	94.70	1518	1520.7	2.7	2	883	0.8702	1
65	3-Methyladipic acid-2TMS	3	61.45	1533	1535	2	2	875	0.6113	1
66	Erythronic acid-4TMS	3	73.98	1542	-	-	3	827	0.7029	1
67	1,3-Dioxolane, 2-isopropyl	6	77.08	1562	798	764	3	846	0.7282	0
68	2-Hydroxyglutaric acid-3TMS	1	77.08	1572	1615	43	3	678	0.6127	1
69	Propanone-2TMS	6	52.23	1594	-	-	3	719	0.6186	1
70	Penicillamine-3TMS	2	88.54	1609	1611	2	2	897	0.6481	1
71	Glutamic acid-3TMS	2	83.20	1615	1629	14	3	744	0.6348	1
72	L-Phenylalanine-2TMS	2	86.45	1622	-	-	3	709	0.6116	1
73	6-Deoxy-hexose-4(O-TMS)	4	77.60	1620	-	-	3	788	0.6248	1
74	Benzeneacetic acid, 3-hydroxy-2TMS	3	83.58	1636	1600	36	3	815	0.5417	0
75	D-Arabinopyranose-4TMS	4	92.91	1640	1636.5	3.5	2	904	0.4484	0
76	Dodecanoic acid-TMS	1	99.60	1651	1651	0	2	894	0.6371	1

77	D-Ribopyranose-4TMS	4	94.02	1662	1658	4	2	882	0.7426	1
78	D-Ribopyranose-4TMS	4	52.98	1668	1641.1	26.9	2	803	0.5214	1
79	6-Deoxy-hexose-4(O-TMS)	4	38.00	1686	-	-	3	844	0.5558	1
80	Mercaptoacetic acid-2TMS	3	95.89	1698	-	-	3	669	0.9197	0
81	α-D-Xylopyranose-4(O-TMS)	4	92.53	1707	1729	22	2	871	0.4216	1
82	Penicillamine-3TMS	1	82.29	1711	-	-	3	822	0.3732	1
83	1,3-Dioxolane-2-methanol	6	27	1726	-	-	3	917	0.4141	1
84	Octanoic acid-TMS	1	71.80	1748	1260	488	3	718	0.7436	1
85	D-Allofuranose-5TMS	4	64.10	1811	1852	41	2	788	0.5849	1
86	D-Fructopyranose-5TMS	4	98.80	1805	1836	31	2	783	0.6388	1
87	D-Tagatofuranose-5TMS	4	49.60	1795	1800	5	3	683	0.4779	1
88	Tetradecanoic acid-TMS	1	78.30	1809	1842	33	3	704	0.6207	1
89	2-Hexenedioic acid-2TMS	1	40.60	1852	-	-	3	707	0.2959	1
90	D-Glucose-5(O-TMS)	4	93.28	1850	1930	80	3	880	0.7331	1
91	Gluconic acid, 2-methoxime-5TMS	3	27.90	1875	-	-	3	715	0.2755	0
92	α-Ketobutyric acid-TMS	1	90.60	1883	989	894	3	743	0.7595	1
93	β-D-Talopyranose-5TMS	4	40.67	1895	1895.5	0.5	2	887	0.7985	0
94	Pentadecanoic acid-TMS	1	99.60	1909	1942	33	3	868	0.8909	1
95	Pentadecanoic acid-TMS	1	99.60	1917	1942	25	2	881	0.8465	1
96	Hydrocaffeic acid-3TMS	3	52.20	1938	1950	12	3	774	0.1735	1
97	Pentadecanoic acid-TMS	1	98.80	1945	1942	3	2	897	0.9299	1
98	1-Hexadecanol-O-TMS	6	94.40	1958	1965	7	2	886	0.7597	1

99	Palmitic acid-TMS	1	85.07	2006	2039	33	3	769	0.5788	1
100	Ribitol-5TMS	5	67.70	2032	1767	265	-	631	0.6524	1
101	2-(Acetylamino)-2-deoxy-hexose-4(O-TMS)	4	17.53	2087	-	-	3	668	0.4274	0
102	L-Sorbose-5TMS	4	67.50	2111	1866.6	244.4	-	474	0.3074	1
103	Heptadecanoic acid-TMS	1	16.70	2142	2140	2	2	871	0.4000	1
104	4-[1-(4-Methoxyphenyl)-1-methylethyl]phenoxy)silane-TMS	6	31.70	2204	-	-	3	860	0.6208	0
105	Butanoic acid, 3-methyl-2-oxo-TMS	1	18.7	2273	-	-	3	783	0.6496	0
106	Linoleic acid-TMS	1	38.00	2265	2201	64	3	936	0.7137	1
107	Glyceryl-glycoside-TMS-ether	4	34.70	2291	-	-	3	720	0.4170	1
108	Trans-9-octadecenoate-TMS	6	78.30	2299	2215	84	3	804	0.5672	1
109	2-oxo-propanoate-TMS	1	66.66	2323	-	-	3	825	0.6696	0
110	Oleamide	6	37.30	2364	2228	136	3	833	0.8024	1
111	Myo-Inositol-phosphate-2TMS-5(O-TMS)	5	85.40	2400	-	-	3	687	0.5204	1
112	Uridine, 2',3',5'-tris-O-TMS	6	59.30	2445	2444	1	-	594	0.7492	1
113	9,12,15-Octadecatrienoic acid-methyl-ethyl ester-2(O-TMS)	6	58.20	2617	-	-	-	556	0.5313	0
114	Docosanoic acid-TMS	1	94.70	2636	2638	2	2	792	0.6911	1
115	Hexadecamethylheptasiloxane	6	91.70	2691	1553	1138	-	447	0.7767	0
116	Oleic acid propyl ester-2(O-TMS)	6	44.00	2750	2784	34	3	760	0.5777	0

117	Carbostyril, 3-ethyl-4-hydroxy-7-methoxy-	6	23.80	2777	-	-	3	659	0.3856	0
118	Dimethyl-(2-methylpropoxy)-ane-TMS	6	80.00	2773	-	-	3	666	0.5673	0
119	Tetracosanoic acid-TMS	1	91.60	2834	2829	5	3	755	0.4399	1
120	2,2,7,7-Tetramethyl-4,5-diphenyl-3,6-dioxa-2,7-disilaoctane	6	31.34	2879	-	-	3	734	0.3244	0
121	Cholesteryl chloroformate	6	48.8	2895	-	-	3	743	0.7166	0
122	cis-5,8,11,14,17-Eicosapentaenoic acid-TMS	1	89.50	2992	2380	612	3	787	0.5665	1
123	Coprostan-3-ol-TMS	5	95.8	3067	-	-	3	815	0.8015	1
124	α-Tocopherol-O-TMS	6	98.10	3131	3156	25	-	581	0.7539	1
125	Ergost-8(14)-ene	6	98.10	3153	-	-	3	835	0.7869	0
126	Ethyl iso-allocholate	6	91.6	3257	-	-	-	609	0.7768	1
127	Pregnanediol	5	91.66	3263	2763	500	3	681	0.7132	1
128	Deoxycholic acid-3TMS	3	17.90	3278	3012	266	3	768	0.2670	1
129	2-Pentanol-TMS	6	36.4	3452	833.1	2618.9	3	811	0.6068	1
130	1-(1,3-Dioxolan-2-yl)acetone	6	76.11	1147	-	-	3	694	0.8794	0
131	(R)-3-Hydroxybutyric acid	1	89.50	1161	1147.9	13.1	3	651	0.6817	1
132	β-Amino isobutyric acid	3	87.60	1170	1148	22	3	729	0.8135	1
133	2-Hydroxy-3-methylvaleric acid-2TMS	1	69.90	1240	1253	13	3	718	0.7901	1
134	Octanoic acid-TMS	1	97.70	1264	1260	4	2	910	0.7866	1
135	Glyceric acid-3TMS	1	89.50	1329	1350	21	3	749	0.7670	1

136	Hexanoic acid-TMS	1	73.90	1359	1071	288	3	747	0.7033	1
137	Aziridine-TMS	6	59.37	1362	-	-	3	773	0.4902	1
138	L-Aspartic acid-3TMS	2	83.30	1468	1502.4	34.4	-	532	0.6890	1
139	Oxalic acid-2TMS	3	87.50	1552	1124	428	3	752	0.8401	1
140	1-[2-(Isobutyryloxy)-1-methylethyl]-2,2-dimethylpropyl 2-methylpropanoate	6	98.80	1587	-	-	3	888	0.9835	0
141	β-Phenyllactic acid-2TMS	3	78.10	1579	1582	3	2	834	0.7291	1
142	D-Arabinopyranose-4TMS	4	93.70	1644	1636.5	7.5	3	654	0.6894	0
143	β-D-Xylopyranose-4TMS	4	82.40	1757	1754.8	2.2	2	897	0.4740	0
144	Azelaic acid-2TMS	3	88.54	1791	1787	4	-	602	0.7894	1
145	D-Fructopyranose-5TMS	4	89.50	1814	1836	22	3	669	0.5897	0
146	N-Acetyl-D-glucosamine-4TMS	4	78.10	1950	2091	141	3	763	0.5883	1
147	Myo-Inositol-6TMS	5	92.50	2080	2153	73	3	866	0.6215	1
148	Diethylhexyl adipate	6	100.00	2388	2382	6	2	920	1.0357	1
149	19,12-Octadecadiynoic acid-TMS	3	58.3	3273	2311	962	-	562	0.4984	1
