# Supplementary material for: Human Fecal Metabolome Reflects Differences in Body Mass Index, Physical Fitness, and Blood Lipoproteins in Healthy Older Adults
Source: Metabolites. 2021 Oct 21;11(11):717. doi: 10.3390/metabo11110717 (PMC8620003; doi:10.3390/metabo11110717)
Supplement: Supplementary file 1 [file metabolites-11-00717-s001.zip › Table S2.pdf]

Table S2. The results from the ANOVA analysis performed on NMR (A) and GC-MS (B) based metabolite data to investigate the differences between low fitness and high fitness groups including all subjects. (HF = high fitness, LF = low fitness).

(A) NMR metabolites

No.	Metabolites	*p*-value	Effect size (%)	Fold change (HF/LF)
1	leucine	0.0264	3.02	0.8736
2	isoleucine	0.0470	2.42	0.8816
3	valine	0.0306	2.87	0.8750
4	propionic acid	0.0083	4.25	0.8569
5	lactic acid	0.0316	2.83	0.7346
6	alanine	0.0281	2.96	0.8704
7	methionine	0.0084	4.23	0.7828
8	glutamic acid	0.0231	3.16	0.7473
9	sarcosine	0.0490	2.37	0.8628
10	threonine	0.0266	3.02	0.8607
11	phenylalanine	0.0454	2.46	0.8881

(B) GC-MS metabolites

No.	Metabolites	*p*-value	Effect size (%)	Fold change (HF/LF)
1	1,4,5,6-tetrahydropyridazine	0.0453	2.91	1.1506
2	Oxalic acid-2TMS	0.0173	4.09	1.2623
3	Glycine-2TMS	0.0401	3.06	1.5645
4	Vinyl acetate	0.0423	3.00	1.9223
5	6-Azathiothymine	0.0139	4.36	4.0077
6	Cyclohexanecarboxylic acid-TMS	0.0455	2.91	2.6325
7	N-[4-(Dimethylamino)butyl]-N-methylacetamide	0.0053	5.58	2.0984
8	Oxalic acid-2TMS	0.0021	6.75	3.0641
9	Nicotinic acid-TMS	0.0320	3.34	1.3830
10	Propanedioic acid-2TMS	0.0487	2.83	1.2990
11	2,2-Dimethyl-3-hydroxybutanoic acid-2TMS	0.0336	3.27	2.7446
12	Glutamic acid-3TMS	0.0496	2.80	1.6258
13	α-D-Xylopyranose-4(O-TMS)	0.0485	2.83	2.2934
14	Penicillamine-3TMS	0.0217	3.81	3.7572
15	Ethyl iso-allocholate	0.4330	2.97	0.6974
16	2-Pentanol-TMS	0.0324	3.32	0.5719
17	β-D-Xylopyranose-4TMS	0.0133	4.20	2.0207
