# Supplementary material for: Human Fecal Metabolome Reflects Differences in Body Mass Index, Physical Fitness, and Blood Lipoproteins in Healthy Older Adults
Source: Metabolites. 2021 Oct 21;11(11):717. doi: 10.3390/metabo11110717 (PMC8620003; doi:10.3390/metabo11110717)
Supplement: Supplementary file 1 [file metabolites-11-00717-s001.zip › Table S3.pdf]

Table S3. The results from the ANOVA analysis performed on the sex stratified NMR and GC-MS based metabolite data to investigate the differences between low fitness (LF) and high fitness (HF) groups.

Males NMR Metabolites

No.	Metabolites	*p*-value	Effect size (%)	Fold change (HF/LF)
1	methionine	0.0134	7.32	0.7498
2	threonine	0.014	7.23	0.8049

Females NMR Metabolites

No.	Metabolites	*p*-value	Effect size (%)	Fold change (HF/LF)
1	propionic acid	0.0393	5.33	0.8298
2	malic acid	0.0048	9.75	0.6775
3	fumaric acid	0.0019	11.69	0.7865

Males GC-MS Metabolites

No.	Metabolites	*p*-value	Effect size (%)	Fold change (HF/LF)
1	Vinyl acetate	0.0066	10.64	5.0015
2	6-Azathiothymine	0.0328	6.71	9.4809
3	2-Hydroxyisovaleric acid-2TMS	0.0298	6.95	2.5287
4	2-Hydroxyisocaproic acid-TMS	0.0458	5.91	2.4201
5	Oxalic acid-2TMS	0.0220	7.69	3.0843
6	Penicillamine-3TMS	0.0270	7.19	2.4151
7	6-Deoxy-hexose-4(O-TMS)	0.0487	5.76	1.7455
8	D-Arabinopyranose-4TMS	0.0316	6.81	4.2161
9	D-Ribopyranose-4TMS	0.0283	7.08	2.7225
10	Penicillamine-3TMS	0.0268	7.21	7.6182
11	Glyceryl-glycoside-TMS-ether	0.0428	6.07	3.2981
12	α-Tocopherol-O-TMS	0.0491	5.74	0.5411
13	Ethyl iso-allocholate	0.0203	7.89	0.5838

Females GC-MS Metabolites

No.	Metabolites	*p*-value	Effect size (%)	Fold change (HF/LF)
1	Carbodiimide-2TMS	0.0460	5.73	1.2327
2	1,4,5,6-tetrahydropyridazine	0.0087	9.70	1.3307
3	Oxalic acid-2TMS	0.0081	9.86	1.4216
4	N-[4-(Dimethylamino)butyl]-N-methylacetamide	0.0369	6.25	2.2540
5	Oxalic acid-2TMS	0.0371	6.23	2.9898
6	Glutaric acid-2TMS	0.0434	5.86	1.8114
7	Dimethyl-(2-methylpropoxy)-ane-TMS	0.0382	6.16	0.4503
8	β-D-Xylopyranose-4TMS	0.0463	5.71	2.3369
