# Supplementary material for: Human Fecal Metabolome Reflects Differences in Body Mass Index, Physical Fitness, and Blood Lipoproteins in Healthy Older Adults
Source: Metabolites. 2021 Oct 21;11(11):717. doi: 10.3390/metabo11110717 (PMC8620003; doi:10.3390/metabo11110717)
Supplement: Supplementary file 1 [file metabolites-11-00717-s001.zip › Table S4.pdf]

Table S4. The results from the ANOVA analysis performed on NMR based metabolite data to investigate the differences between low BMI and high BMI groups including all subjects. MH = mean high BMI, ML = mean low BMI.

No.	Metabolites	*p*-value	Effect size (%)	Fold change (MH/ML)
1	leucine	0.0032	7.83	1.2492
2	isoleucine	0.0143	5.48	1.2102
3	valine	0.0049	7.15	1.2391
4	propionic acid	0.0367	4.01	1.1678
5	alanine	0.0014	9.15	1.2754
6	methionine	0.0352	4.08	1.2688
7	glutamic acid	0.0179	5.13	1.4895
8	pyruvic acid	0.0300	4.33	1.5769
9	lysine	0.0091	6.19	1.2376
10	glycine	0.0076	6.47	1.2030
11	uracil	0.0067	6.66	1.2345
12	tyrosine	0.0232	4.72	1.2047
13	phenylalanine	0.0026	8.17	1.2588
14	xanthine	0.0078	6.41	1.3692
