# Supplementary material for: Human Fecal Metabolome Reflects Differences in Body Mass Index, Physical Fitness, and Blood Lipoproteins in Healthy Older Adults
Source: Metabolites. 2021 Oct 21;11(11):717. doi: 10.3390/metabo11110717 (PMC8620003; doi:10.3390/metabo11110717)
Supplement: Supplementary file 1 [file metabolites-11-00717-s001.zip › Table S5.pdf]

Table S5. The results from the ANOVA analysis performed on the sex stratified NMR based metabolite data to investigate the differences between low BMI and high BMI groups**.**

(A) Males

No.	Metabolites	*p*-value	Effect size (%)	Fold change (MH/ML)
1	leucine	0.0124	12.09	1.2830
2	isoleucine	0.0138	11.75	1.3021
3	valine	0.0156	11.35	1.2782
4	alanine	0.0047	15.21	1.3273
5	methionine	0.0319	9.06	1.3767
6	lysine	0.0064	14.21	1.4014
7	glycine	0.0049	15.06	1.2826
8	threonine	0.0188	10.76	1.2915
9	uracil	0.0092	13.04	1.3120
10	tyrosine	0.0166	11.17	1.3117
11	phenyalanine	0.0038	15.84	1.3601
12	xanthine	0.0307	9.18	1.4788

(B) Females

No.	Metabolites	*p*-value	Effect size (%)	Fold change (MH/ML)
1	glutamic acid	0.0429	7.21	1.6495
2	malic acid	0.0236	8.82	1.4523
3	fumaric acid	0.0219	9.04	1.4487
