# Supplementary material for: Human Fecal Metabolome Reflects Differences in Body Mass Index, Physical Fitness, and Blood Lipoproteins in Healthy Older Adults
Source: Metabolites. 2021 Oct 21;11(11):717. doi: 10.3390/metabo11110717 (PMC8620003; doi:10.3390/metabo11110717)
Supplement: Supplementary file 1 [file metabolites-11-00717-s001.zip › Table S6.pdf]

Table S6. The Pearson's correlation coefficients and *p*-values calculated between the 36 fecal metabolites and the eight lipoproteins including total *chol*, HDL*chol*, LDL*chol*, VLDL*chol*, total *chol*/HDL*chol*, total *chol*/HDL-Apo A, LDL*chol*/HDL*chol* and VLDL*chol*/HDL*chol*, including all subjects. *Significant correlations.

Metabolites	coeff.	total *chol*	HDL *chol*	LDL *chol*	VLDL *chol*	total *chol*/ HDL*chol*	total *chol*/ HDL-Apo A	LDL*chol*/ HDL*chol*	VLDL*chol*/ HDL*chol*
valeric acid	r/p	0.04/0.57	0.1/0.44	0.01/0.44	0.04/0.44	-0.03/0.67	-0.08/0.29	-0.02/0.82	0.02/0.76
isovaleric acid	r/p	0.1/0.19	-0.04/0.59	0.1/0.21	**0.15/0.05***	0.14/0.08	0.005/0.95	0.14/0.08	**0.16/0.04***
leucine	r/p	-0.09/0.24	-0.00/0.99	-0.08/0.32	0.08/0.32	-0.05/0.54	-0.14/0.07	-0.05/0.5	0.07/0.35
isoleucine	r/p	-0.12/0.11	0.02/0.81	-0.12/0.13	0.06/0.42	-0.07/0.37	**-0.17/0.03***	-0.08/0.3	0.06/0.42
valine	r/p	-0.07/0.39	0.01/0.89	-0.07/0.39	0.08/0.32	-0.05/0.57	-0.13/0.09	-0.05/0.5	0.07/0.39
propionic acid	r/p	-0.01/0.98	0.06/0.45	0.01/0.89	0.01/0.92	-0.02/0.78	-0.14/0.08	0.003/0.96	0.03/0.71
ethanol	r/p	**-0.19/0.02***	-0.1/0.21	-0.09/0.24	-0.06/0.5	-0.04/0.64	-0.05/0.55	-0.02/0.82	-0.03/0.75
lactic acid	r/p	**-0.2/0.006***	0.003/0.96	-0.14/0.97	-0.09/0.3	-0.13/0.1	**-0.16/0.046***	-0.1/0.18	-0.06/0.43
alanine	r/p	-0.1/0.19	0.02/0.83	-0.09/0.28	0.007/0.9	-0.08/0.32	-0.1/0.22	-0.08/0.34	-0.002/0.9
butyric acid	r/p	-0.04/0.62	**0.15/0.05***	-0.06/0.42	-0.08/0.3	-0.12/0.12	**-0.15/0.05***	-0.1/0.2	-0.08/0.31
acetic acid	r/p	-0.06/0.4	0.09/0.27	-0.04/0.64	-0.07/0.4	-0..1/0.21	-0.13/0.09	-0.06/0.43	-0.06/0.44
methionine	r/p	-0.04/0.66	0.09/0.26	-0.07/0.36	0.1/0.16	-0.1/0.23	**-0.17/0.026***	-0-1/0.19	0.06/0.48
acetone	r/p	0.07/0.36	-0.01/0.85	0.06/0.43	0.03/0.72	0.05/0.51	0.02/0.84	0.05/0.52	0.02/0.82
glutamic acid	r/p	-0.1/0.18	0.08/0.33	-0.08/0.28	-0.06/0.5	-0.14/0.08	-0.11/0.16	-0.11/0.18	-0.07/0.39
pyruvic acid	r/p	-0.1/0.18	0.02/0.84	-0.07/0.38	-0.1/0.19	-0.1/0.2	-0.08/0.29	-0.08/0.29	-0.09/0.25
aspartic acid	r/p	-0.07/0.38	0.06/0.45	-0.09/0.26	0.09/0.28	-0.09/0.28	-0.09/0.28	-0.1/0.22	0.07/0.39
sarcosine	r/p	-0.06/0.45	0.003/0.96	-0.05/0.5	0.009/0.9	-0.03/0.71	-0.12/0.12	-0.03/0.67	0.018/0.82
trimethylamine	r/p	-0.02/0.79	-0.005/0.9	-0.01/0.87	0.13/0.11	0.015/0.85	-0.016/0.84	0.019/0.8	0.12/0.14
lysine	r/p	-0.14/0.07	0.00/0.99	-0.1/0.18	-0.04/0.7	-0.08/0.32	**-0.15/0.05***	-0.07/0.35	-0.01/0.89
malonic acid	r/p	0.06/0.44	0.04/0.63	0.05/0.51	0.03/0.67	-0.009/0.9	-0.002/0.97	0.002/0.97	0.01/0.89
methanol	r/p	-0.04/0.6	0.12/0.12	-0.06/0.45	-0.05/0.5	-0.14/0.08	-0.14/0.08	-0.11/0.15	-0.08/0.29
carnitine	r/p	-0.04/0.65	0.12/0.12	-0.05/0.55	-0.09/0.3	-0.1/0.17	-0.02/0.82	-0.09/0.27	-0.09/0.26
glycine	r/p	-0.07/0.38	0.02/0.8	-0.08/0.32	0.057/0.55	-0.05/0.5	-0.14/0.08	-0.06/0.42	-0.04/0.63
glycerol	r/p	-0.06/0.44	0.13/0.11	-0.09/0.27	-0.09/0.3	-0.12/0.12	**-0.15/0.05***	-0.11/0.17	-0.11/0.17
guanidinoacetic acid	r/p	0.05/0.55	0.15/0.06	-0.01/0.86	-0.06/0.5	-0.08/0.33	-0.09/0.26	-0.08/0.36	-0.1/0.23
betaine	r/p	-0.07/0.34	0.05/0.55	-0.1/0.2	0.11/0.14	-0.07/0.35	-0.13/0.09	-0.17/0.24	0.1/0.24
threonine	r/p	-0.13/0.09	0.07/0.36	-0.1/0.19	0.03/0.66	-0.1/0.18	**-0.18/0.024***	-0.09/0.29	0.04/0.63
malic acid	r/p	0.05/0.54	-0.470.6	0.04/0.61	0.09/0.25	0.09/0.24	-0.06/0.46	0.08/0.33	**0.11/0.048***
glucose	r/p	-0.03/0.68	0.1/0.2	-0.04/0.62	-0.07/0.4	-0.1/0.23	-0.03/0.72	-0.07/0.35	-0.07/0.34
galactose	r/p	0.02/0.78	0.13/0.09	-0.003/0.9	-0.02/0.8	-0.07/0.34	-0.03/0.53	-0.05/0.53	-0.06/0.46
uracil	r/p	-0.11/0.16	0.08/0.31	-0.07/0.37	-0.05/0.5	0.14/0.08	**-0.18/0.02***	-0.09/0.24	-0.06/0.41
fumaric acid	r/p	0.05/0.5	-0.03/0.72	0.03/0.66	0.09/0.23	0.07/0.35	-0.08/0.33	0.06/0.48	0.09/0.25
tyrosine	r/p	-0.06/0.44	0.04/0.57	-0.07/0.4	0.05/0.52	-0.06/0.41	-0.09/0.23	-0.07/0.4	-0.04/0.57
phenylalanine	r/p	-0.13/0.1	0.01/0.86	-0.11/0.18	0.04/0.61	-0.08/0.33	**-0.16/0.04***	-0.08/0.34	0.04/0.6
xanthine	r/p	-0.09/0.24	0.00/0.99	-0.03/0.66	-0.09/0.2	-0.06/0.41	-0.13/0.1	-0.03/0.7	-0.07/0.36
formic acid	r/p	**-0.19/0.02***	-0.13/0.11	-0.1/0.2	-0.04/0.6	-0.02/0.83	-0.08/0.32	-0.024/0.8	-0.00/0.99
