# Supplementary material for: Human Fecal Metabolome Reflects Differences in Body Mass Index, Physical Fitness, and Blood Lipoproteins in Healthy Older Adults
Source: Metabolites. 2021 Oct 21;11(11):717. doi: 10.3390/metabo11110717 (PMC8620003; doi:10.3390/metabo11110717)
Supplement: Supplementary file 1 [file metabolites-11-00717-s001.zip › Table S7.pdf]

Table S7. The thresholds and numbers of subjects representing top 1/3 and bottom 1/3 according to the selected eight lipoproteins including total *chol*, HDL*chol*, LDL*chol*, VLDL*chol*, total *chol*/HDL*chol*, total *chol*/HDL-Apo A, LDL*chol*/HDL*chol* and VLDL*chol*/HDL*chol*.

Lipoprotein variables	Class	Thresholds	Number of Subjects
total *chol*(mmol/L)	high 1/3	≥6.2	51
	low 1/3	≤5.1	49
HDL*chol*(mmol/L)	high 1/3	≥2.1	54
	low 1/3	≤1.58	54
LDL*chol*(mmol/L)	high 1/3	≥3.6	50
	low 1/3	≤2.6	54
VLDL*chol*(mmol/L)	high 1/3	≥0.8	40
	low 1/3	≤0.4	54
total *chol*/HDL*chol*	high 1/3	≥3.6	54
	low 1/3	≤2.65	54
total *chol*/HDL-Apo A	high 1/3	≥0.96	55
	low 1/3	≤0.72	54
LDL*chol*/HDL*chol*	high 1/3	≥2.22	54
	low 1/3	≤1.29	54
VLDL*chol*/HDL*chol*	high 1/3	≥0.4	55
	low 1/3	≤0.23	54
tg	high 1/3	≥1.49	55
	low 1/3	≤0.98	53
